# Combinatorial Methylerythritol Phosphate Pathway Engineering and Process Optimization for Increased Menaquinone-7 Synthesis in *Bacillus subtilis*

**DOI:** 10.4014/jmb.1912.12008

**Published:** 2020-02-18

**Authors:** Taichi Chen, Hongzhi Xia, Shixiu Cui, Xueqin Lv, Xueliang Li, Yanfeng Liu, Jianghua Li, Guocheng Du, Long Liu

**Affiliations:** 1 Key Laboratory of Carbohydrate Chemistry and Biotechnology, Ministry of Education, Jiangnan University, Wuxi 2422, P.R. China; 2 Key Laboratory of Industrial Biotechnology, Ministry of Education, Jiangnan University, Wuxi 141, P.R. China; 3 Richen Bioengineering Co., Ltd., Nantong 226000, P.R. China

**Keywords:** Menaquinone, methylerythritol phosphate pathway, *Bacillus subtilis*, process optimization

## Abstract

Vitamin K2 (menaquinone) is an essential vitamin existing in the daily diet, and menaquinone-7 (MK- 7) is an important form of it. In a recent work, we engineered the synthesis modules of MK-7 in *Bacillus subtilis*, and the strain BS20 could produce 360 mg/l MK-7 in shake flasks, while the methylerythritol phosphate (MEP) pathway, which provides the precursor isopentenyl diphosphate for MK-7 synthesis, was not engineered. In this study, we overexpressed five genes of the MEP pathway in BS20 and finally obtained a strain (BS20DFHG) with MK-7 titer of 415 mg/l in shake flasks. Next, we optimized the fermentation process parameters (initial pH, temperature and aeration) in an 8-unit parallel bioreactor system consisting of 300-ml glass vessels. Based on this, we scaled up the MK-7 production by the strain BS20DFHG in a 50-l bioreactor, and the highest MK-7 titer reached 242 mg/l. Here, we show that the engineered strain BS20DFHG may be used for the industrial production of MK-7 in the future.

## Introduction

Vitamin K2 (menaquinone, MK) is a series of compounds that share the 2-methyl-1,4-naphthoquinone but with different lengths and saturation degrees of the polyisoprene side chain attached to the 3-position. Among these compounds is menaquinone-7 (MK-7), which is the term for 2-methyl-3-heptaprenyl-1,4-naphthoquinone, meaning the polyisoprene side chain consists of seven prenyl units [1]. MK-7 can be directly absorbed and utilized by the human body and has a longer half-life in human blood than other forms of vitamin K [2]. Addition of MK-7 in the daily diet can prevent bone fractures [2], treat vitamin K deficiency hemorrhagic disease [3], or prevent arterial calcification [4]. The biologically active MK-7 is mainly produced by microbial fermentation, including solid-state fermentation and liquid fermentation [5, 6]. The solid-state fermentation takes natto (fermented soybean) as the substrate, and the reported maximum yield is 67 mg/kg by *Bacillus subtilis natto* [7]. The parameters of solid-state fermentation are challenging to control, and a large amount of metabolic heat is generated, resulting in the slow growth of bacteria and low MK-7 production. Therefore, increasing attention is being paid to the production of MK-7 by liquid fermentation, and the strains used in this method include *Bacillus subtilis natto*, *Bacillus subtilis licheniformis*, *Flavobacterium*, etc. [8-10]. Previous studies have explored the effects of biofilm growth, dissolved oxygen changes, pH and electron generation on the production of MK-7 [2,11-13]. In recent work, we engineered the synthesis pathway of MK-7 in *B. subtilis* using a quorum-sensing system, and the highest titer of MK-7 reached 360 mg/l in shake flasks and 200 mg/l in the 15-l bioreactor [14].

**Fig. 1:**
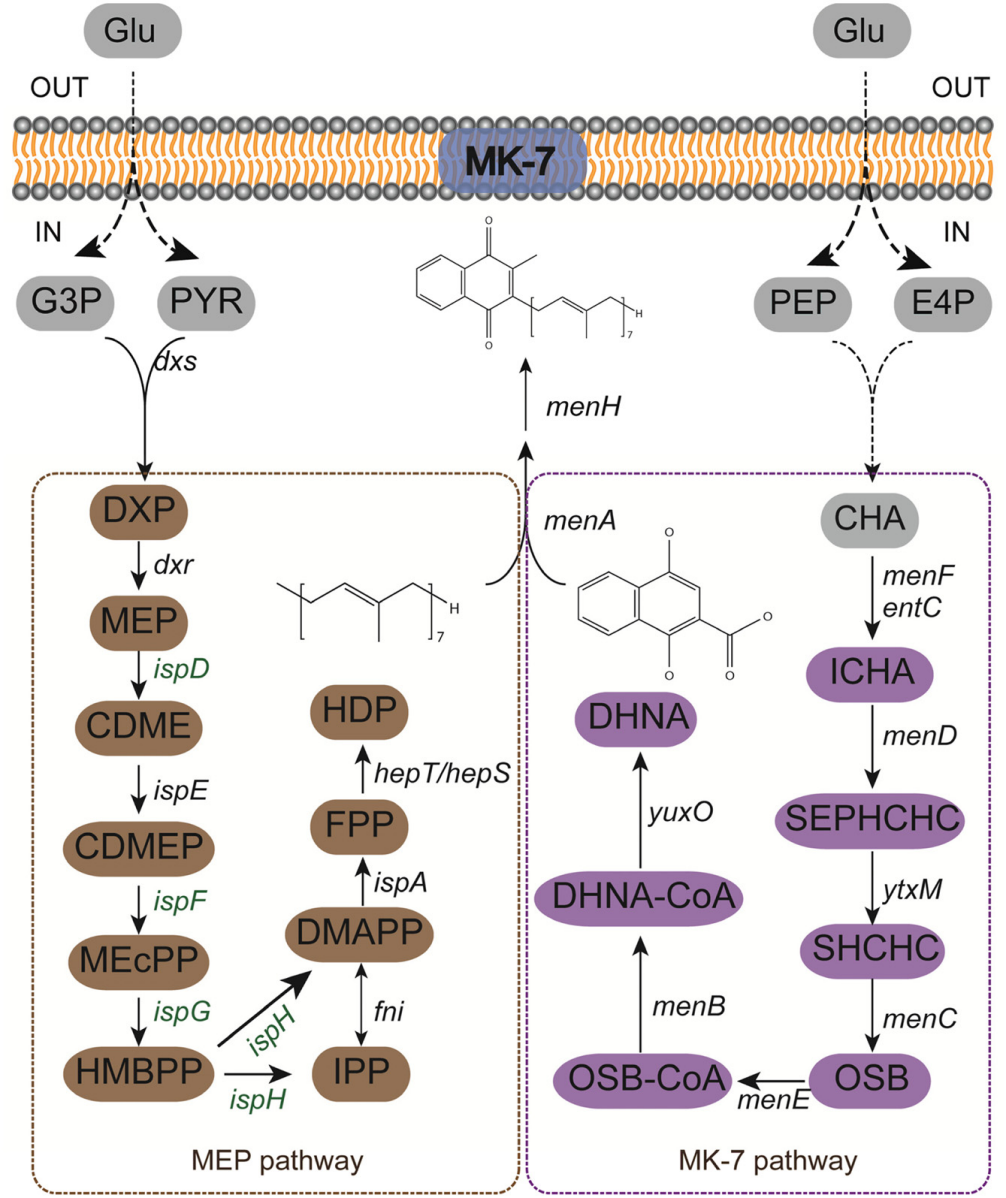
The pathway of MK-7 biosynthesis. MK-7 is synthesized from two precursors that come from MEP pathway and MK-7 pathway, respectively. The dashed lines mean multiple reactions and the genes in green are the overexpressed genes in the strain BS20DFHG. **Enzymes:** Dxs, 1-deoxyxylulose-5-phosphate synthase; Dxr, 1-deoxyxylulose-5-phosphate reductoisomerase; IspD, 2-C-methylerythritol 4-phosphate cytidylyltransferase; IspE, 4-diphosphocytidyl-2-C-methylerythritol kinase; IspF, 2-C-methylerythritol 2,4-cyclodiphosphate synthase; IspG, 4-hydroxy-3-methylbut-2-enyl diphosphate synthase; IspH, 4-hydroxy-3-methylbut-2-enyl diphosphate reductase; Fni, isopentenyl diphosphate isomerase; IspA, farnesyl diphosphate synthase; HepS/HepT, heptaprenyl diphosphate synthase component I/II. MenF, isochorismate synthase; EntC, isochorismate synthase from *E. coli* K12; MenD, 2-succinyl-5-enolpyruvyl-6-hydroxy-3-cyclohexene-1-carboxylate synthase; YtxM, 2-succinyl-6-hydroxy-2,4-cyclohexadiene-1-carboxylate synthase; MenC, o-succinylbenzoate synthase; MenE, o-succinylbenzoic acid-CoA ligase; MenB, 1,4-dihydroxy-2-naphthoyl-CoA synthase; YuxO, 1,4-dihydroxy-2-naphthoyl-CoA hydrolase; MenA, 1,4-dihydroxy-2-naphthoate heptaprenyltransferase; MenH, demethylmenaquinone methyltransferase. Abbreviation of metabolites: Glu, glucose; PEP, phosphoenolpyruvate; PYR, pyruvate; E4P, erythrose 4-phosphate; G3P, glyceraldehyde-3-phosphate; CHA, Chorismite; ICHA, isochorismate; SEPHCHC, 2-succinyl-5-enolpyruvyl-6-hydroxy-3-cyclohexene-1-carboxylate; SHCHC, 2-succinyl-6-hydroxy-2,4-cyclohexadiene-1-carboxylate; OSB, 2-succinylbenzoate; OSB-CoA, 2-succinyl benzoyl-CoA; DHNA-CoA, 1,4-dihydroxy-2-naphthoyl-CoA; DHNA, 1,4-dihydroxy-2-naphthoate; DXP, 1-deoxyxylulose-5-phosphate; MEP, methyl-erythritol-4-diphosphate; CDME, methylerythritol cytidyl diphosphate; CDMEP, 4-diphosphocytidyl-2-C-methyl-D-erythritol-2-phosphate; MEcPP, 2-C-methyl-D-erythritol-2,4-cyclodiphosphate; HMBPP, 1-hydroxy-2-methyl-2-butenyl 4-diphosphate; DMAPP, dimethylallyl diphosphate; IPP, isopentenyl diphosphate; FPP, farnesyl diphosphate; HDP, heptaprenyl diphosphate; MK-7, menaquinone-7.

*B. subtilis* is Generally Recognized as Safe (GRAS) and is a typical gram-positive bacterium. The biosynthesis of MK-7 precursors in *B. subtilis* can be divided into two modules (Fig. 1). Naphthoquinone, derived from chorismic acid, is the bone structure of MK-7. The polyisoprene side chain is synthesized from glyceraldehyde-3-phosphate (G3P) and pyruvate (PYR) through the MEP pathway. Then, the polyisoprene side chain will be joined to the naphthoquinone to form demethylmenaquinone (DMK) by 1, 4-dihydroxy-2-naphthoate octaprenyltransferase (MenA). Finally, MK-7 is synthesized when the methylation of DMK is finished. In other recent work, we developed a bifunctional quorum-sensing system in *B. subtilis* 168 to engineer the synthesis modules of MK-7, and obtained a recombinant strain BS20 [14], while the MEP pathway had not been engineered. There are many studies about the MEP pathway and its final product, isopentenyl diphosphate (IPP). The accumulation of IPP can increase the production of many natural products such as menaquinone, ubiquinone, carotenoids and taxadiene [15-19]. 1-deoxyxylulose-5-phosphate synthase (Dxs) and 1-deoxyxylulose-5-phosphate reductoisomerase (Dxr) are thought to catalyze rate-limiting steps of the MEP pathway. Up-regulating Dxs and Dxr in *B. subtilis* can significantly improve the production of MK-7 [5]. The titer of carotenoids in *B. subtilis* was increased by engineering the five enzymes (IspD - IspH) catalyzing MEP to IPP and dimethylallyl diphosphate (DMAPP) [16].

In this study, we focused on enhancing the transcription level of five *isp*-genes (*ispD, ispE, ispF, ispH*, and *ispG*) in the MEP pathway to promote the production of MK-7. The highest MK-7 production of 415 ± 3.2 mg/l was observed in shake culture of the engineered strain BS20DFHG. Then, we conducted fermentation optimization in an 8-unit, 300-ml bioreactor system, and the highest titer of MK-7 was 185.57 ± 6.3 mg/l at 41oC, 2 vvm and an initial pH of 7.0. Finally, we conducted fermentation in a 50-l bioreactor under optimized conditions, and 242 ± 5.5 mg/l of MK-7 was produced.

## Materials and Methods

### Microorganisms and Reagents

All the constructed *B. subtilis* strains and plasmids in this study are listed in Table 1. All microorganisms were cultivated in Luria–Bertani (LB) liquid culture or on LB agar plates at 37°C for genetic experiments. The fermentation medium for shake flasks consisted of 5% (w/v) glucose, 5% (w/v) soy peptone, 5%(w/v) sucrose and 0.06% (w/v) KH_2_PO_4_. Culture media were sterilized for 20 min at 115°C. Reagents mentioned above were purchased from Sinopharm Chemical Reagent Co., Ltd. (Shanghai, China). Appropriate antibiotics were added into the medium: kanamycin (50 μg/ml), zeocin (20 μg/ml), ampicillin (100 μg/ml), spectinomycin (50 μg/ml), and chloramphenicol (5 μg/ml). All antibiotics were purchased from Sangon Biotech Co., Ltd. (China). A standard sample of MK-7 was purchased from ChromaDex (USA). Methanol, dichloromethane, 2-propanol, and n-hexane were obtained from Sigma-Aldrich (USA).

### In Situ Replacement of Promoter

The expression of *isp*-genes was strengthened by replacing the native promoter with the strong constitutive promoter P_43_. The homogenous arms of *isp*-genes, target sequences and P_43_ promoter sequences were amplified from *B. subtilis*. The modified DNA fragments and plasmids were sequenced by GENEWIZ (USA). Primers used for genetic engineering were listed in Table S1. These three fragments and the lox71–resistance marker–lox66 cassettes were combined by overlapping PCR. The purified PCR products were used to transform competent *B. subtilis* cells by electroporation. The PDG148 plasmid was then transformed into antibiotic-resistant clones to promote the recombination of lox71 and lox66, which evicted the resistance marker cassette as previously described [20]. Finally, the intracellular plasmid PDG148 was lost by incubation at 50°C for 12 h.

### Shake-Flask Culture of the Engineered *B. subtilis*

A ring of engineered *B. subtilis* strains was picked from the plate and inoculated into 3 ml LB at 37°C with shaking at 220 rpm for 6 h in a 15-ml tube. Each 250-ml Erlenmeyer flask contained 20 ml of fermentation medium, which was inoculated with 1 ml of seed liquid and grown at 41°C with shaking at 220 rpm for 6 days. In addition, 500 μl of fermentation broth was taken every 24 h for MK-7 concentration analysis.

### MK-7 Extraction

MK-7 was extracted from the fermentation broth by extracting agent, which was a mixture of 2-propanol and n-hexane (1:2, v/v) in a 4:1 ratio (organic: liquid, v/v). The mixture was vigorously shaken with a vortex mixer for 10 min and then centrifuged at 7,000 ×*g* for 5 min to separate two phases. The organic phase recovering extracted MK-7 was then separated as a fermentation sample for HPLC analysis.

### Analytical Methods

Cell density was determined from the optical density at 600 nm with a spectrophotometer after suitable dilution with deionized water. The glucose concentration in the media was measured by a glucose–glutamate analyzer (SBA-40C; Biology Institute of Shandong Academy of Sciences, China). High-performance liquid chromatography (Agilent 1260, USA) equipped with a photon diode array UV detector was used to measure the MK-7 concentration of fermentation samples with a C18-ODS column (5 μm, 250 × 4.6 mm, Thermo Fisher Scientific, USA) at 40°C. The mobile phase consisting of methanol: dichloromethane (9:1, v/v) was used at a flow rate of 1 ml/min. A wavelength of 254 nm was used for calibration and analysis. The MK-7 calibration curve was linear between 1 mg/l and 100 mg/l (R^2^ = 0.998).

### Quantitative Reverse-Transcriptase PCR (qRT-PCR) Analysis

Cells were harvested from shake-flask cultures and frozen immediately in liquid nitrogen for 10 min on the third day. Total RNA was purified by using the RNAprep Pure Kit (Tiangen Biotech, China) according to the manual. The quantity and purity of RNA were measured by absorbance measurements at 260 and 280 nm using a Nanodrop ND-1000 spectrophotometer (Thermo Scientific, USA). Subsequently, cDNA, which was obtained by reverse transcription of total RNA using a PrimeScript TM RT-PCR Kit (Takara, China), was used as the template for qPCRs. qPCR was performed in a 96-well plate with a total reaction volume of approximately 20 μl using SYBR Premix Ex Taq TM (Takara) according to the manufacturer’s specifications. The reactions were conducted with a LightCycler 480 II Real-time PCR instrument (Roche Applied Science, Mannheim, Germany). The *hbsU* gene was used as the internal standard [21].

### Fed-Batch Culture in 300-ml Bioreactors

MK-7 fermentation by fed-batch culture of BS20DFHG was performed in 300-ml bioreactors (MiniBox, T&J Bioengineering Co., Ltd. (Shanghai, China). The ‘aeration’ was selected as an influencing factor to simulate the industrial process. The fermentation media used for fed-batch culture consisted of 5% (w/v) soy peptone, 0.06%(w/v) KH_2_PO_4_ and initial glucose concentration of 1.3% (w/v) for each bioreactor. The feeding solution contained 500 g/l glucose. Seed culture was cultured in 250-ml shake flasks containing 50 ml of seed medium with shaking at 220 rpm and 37°C for 6 h. The seed culture was inoculated into a 300-ml bioreactor containing 170 ml fermentation medium by sterile injectors. The initial pH was adjusted to 7.0 by ammonium hydroxide, and the temperature was maintained at 41°C. The 100% calibration is achieved by increasing agitation to approximate 600 rpm and increasing airflow to 1 vvm. In fed-batch culture, when the residual glucose concentration fell below 0.7%(w/v), the feeding solution was pumped into the fermenter to restore the glucose concentration to 0.6-1.5 (w/v). The feeding rates were adjusted every 2 h based on the concentration of residual glucose in the fermentation medium.

### Fed-Batch Culture in 50-l Bioreactor

MK-7 fermentation by fed-batch culture of BS20DFHG was performed in 50-l bioreactors at Richen Bioengineering Co., Ltd., Nantong. It was also performed at an initial glucose concentration of 1.3% (w/v). The fermentation medium used for the fed-batch culture consisted of 5% (w/v) soy peptone and 0.06% (w/v) KH_2_PO_4_. The feeding solution contained 50% (w/v) glucose. Seed culture was carried out in 2-L shake flasks containing 700 ml of seed medium with shaking at 220 rpm and 37°C for 8 h. The seed culture was inoculated into a 50-l fermenter containing 32 L fermentation medium. The pH was set at 7.0 in the beginning, and the temperature was maintained at 4°C in the whole process. The 100% calibration was achieved by increasing agitation to 450 rpm and increasing airflow to 1 vvm. In fed-batch culture, when the residual glucose concentration fell below 1% (w/v), the feeding solution was pumped into the fermenter to restore the glucose concentration to 1.2-1.5 (w/v). The feeding rates were adjusted every 2 h based on the concentration of residual glucose in the fermentation medium.

### Cell Growth Kinetic Model

The kinetic parameters of fermentation equations (Eqs) were analyzed by Origin 2020 software. The differential equations were solved and plotted using MATLAB.

### Statistical Analysis

All experiments were independently carried out at least three times, and the results were expressed as mean ± standard deviation (SD). All the data shown were mean values and are based on the recorded data unless otherwise indicated.

## Results and Discussion

### Increased MK-7 Production by MEP Pathway-Strengthened BS20DFHG Strain

To increase the flux of the MEP pathway, we overexpressed *isp*-genes by replacing native promoters with P_43_ promoter stepwise. Then we conducted fermentation of the recombinant strains in 250-ml shake flasks for 6 days because the highest yield of MK-7 was reached on this day (Fig. S1). Moreover, the relative transcriptional level of *isp*-genes was detected on the third day of culture.

**Fig. 2:**
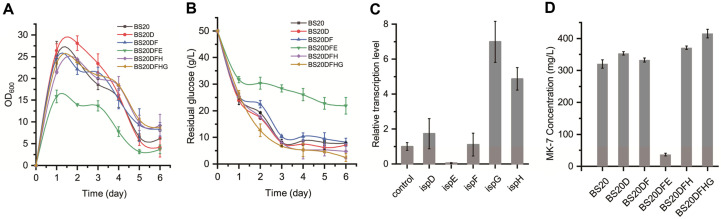
Trends of cell growth, glucose concentration, relative transcription level and MK-7 production in shake flasks. (**A**) Growth curves of engineered strains such as BS20, BS20D, BS20DF, BS20DFE, BS20DFH, and BS20DFHG. (**B**) Glucose concentration of engineered strains during shaking-culture in 250-ml flasks. (**C**) The relative transcriptional levels of *isp*-genes (*ispD, ispF*, *ispH*, and *ispG*) which were overexpressed and the relative transcriptional levels of *ispE* in BS20DFHG, the control gene was *hbsU* from BS20 genome. The transcriptional level of the *isp*-genes in BS20DFHG was the same as it was in the individually enhanced strain, just as the transcriptional level of *ispD* in BS20DFHG was the same as it was in BS20D. (Data shown in Fig. S3) (**D**)The titers of MK-7 synthesized by strains BS20, BS20D, BS20DF, BS20DFE, BS20DFH, and BS20DFHG in 6 days.

First, we replaced the promoter of the gene *ispD* with P_43_ promoter in the genome of BS20, yielding the strain BS20D. Moreover, 2-C-methylerythritol 4-phosphate cytidylyltransferase (IspD) is a phosphocytidyl transferase encoded by *ispD* and is able to couple 2C-methyl-D-erythritol 4-phosphate (MEP) with cytidine triphosphate (CTP). The relative transcriptional level of *ispD* in BS20D was 1.78-times higher than the level of *ispD* in BS20 (Fig. 2C). The optical density (OD_600_) of BS20D rose to 26 in the first two days, and then quickly dropped to 6 on the fifth day (Fig. 2A). At the same time, the increase in OD_600_ value also corresponded to an increase in glucose consumption (Fig. 2B). It was shown that the fermentation titer of MK-7 by BS20D was augmented to 353.2 ± 1.2 mg/l, a 10% increase compared with the production 320.3 ± 2.5 mg/l by BS20 (Fig. 2D). Then we used P_43_ promoter to overexpress *ispF* in the genome of BS20D, yielding strain BS20DF. In addition, 2-C-methylerythritol 2,4-cyclodiphosphate synthase (IspF) is a cyclophosphate synthase encoded by ispF. Although the relative transcriptional level of *ispF* in BS20DF was 1.3 times higher than that of *ispF* in BS20D (Fig. 2C), the fermentation titer of MK-7 by BS20DF was 332.6 ± 3 mg/l. The result showed a 3.9% MK-7 titer increase compared with BS20 but a 5.8% decrease over BS20D (Fig. 2D). This was in contrast to previous research that *ispF* was more important in the flux control of MEP pathway than *ispD* [22]. In their study, Li focused on the systematic analysis of consecutive enzymes in the MEP pathway in *B. subtilis* and successfully increased the production of carotenoids. The yield of carotenoids, which also requires IPP as precursor, showed an 11.38-fold increase after additional insertion of gene *ispF* in *ispD*-overexpressed strain. The reason for the different influence degree of IspF might be that the key points of metabolic flux are usually different because of the difference in different engineered strains [22].

The strain BS20DFE was obtained by overexpressing ispE with P_43_ promoter in BS20DF. Although the growth curve of BS20DFE had the same trend as the other engineered strains (Fig. 2A), the highest OD_600_ value of BSDFE was only 16, and it consumed merely 12 g of glucose in 6 days (Fig. 2B). MEP was speculated to form IspF-MEP complex with IspF in *E. coli* although it might lead to an imbalance in the MEP pathway [23]. If the *ispD*, *ispE*, and *ispF* genes are overexpressed in BS20, the intracellular concentration of IspF may increase, and this might lead to the formation of IspF-MEP complex, resulting in an imbalance in the MEP pathway.

IspG is the term for 4-hydroxy-3-methylbut-2-en-1-yl diphosphate (HMBPP) synthase that is encoded by *ispG*. The overexpression of *ispG* has been reported to promote the production of natural products like lycopene and isoprene that utilize IPP as a biosynthetic precursor [15, 19]. Meanwhile, it was found that activation of the downstream enzyme 4-hydroxy-3-methylbut-2-enyl diphosphate reductase (IspH) could solve the problem of HMBPP accumulation and eliminate the negative effects of *ispG* overexpression [24]. Thus, we decided to overexpress *ispH* before *ispG* to avoid the potential risk of IspG toxicity.

Gene *ispH* was overexpressed by changing the autologous promoter to P_43_ promoter in the strain BS20DF, and the obtained strain was named as BS20DFH. The relative transcriptional level of *ispH* was 4.87-times higher than that of *ispH* in BS20 (Fig. 2C). The overexpression of *ispH* increased MK-7 titer to 370.8 ± 5.2 mg/l (Fig. 2D), showing a 15.8% increase compared with that of BS20. The growth curve of BS20DFH was similar to that of BS20, while the amount and rate of glucose consumption increased (Fig. 2B). Finally, we replaced the promoter of *ispG* with P_43_ promoter in the strain BS20DFH, yielding strain BS20DFHG. Although the strain BS20DFHG did not grow as well as BS20 in the first two days, the OD_600_ of BS20DFHG in the last four days declined slower than BS20 and eventually stayed at 8.8 (Fig. 2A). Thus, we obtained the highest production of MK-7 as 415 ± 3.2 mg/l (Fig. 2D), a 29% increase compared with BS20 and the relative transcriptional level of *ispG* in BS20DFHG was 6.9-times that in BS20. In a previous work, the *dxs, dxr, idi*, and *menA* genes were overexpressed in BS168 to improve the production of MK-7 [5]. In this study, we overexpressed the *isp*-genes of MEP pathway in the BS20 strain to increase the production of MK-7. We supposed that overexpression of *isp*-genes in MEP pathway had a positive effect on the supply of IPP and accumulation of MK-7.

### Optimizing Process Conditions with 300-ml Parallel Bioreactors

To increase the concentration of MK-7 in bioreactors, we designed a set of orthogonal experiments through the software Minitab to optimize the parameters of the fermentation process, including temperature, aeration and initial pH. The orthogonal experimental design is shown in Table 2. The experimental design of the three groups of “1”, “2”, and “3” was selected for each factor. The final fermentation titer of the strain BS20DFHG cultured in 300-ml bioreactors was used as the evaluation index. The experimental design and results are shown in Table 3. From the analysis of the extreme difference, the impact intensity of three factors on the fermentation production of MK-7 was C (initial pH) >A(temperature)>B (aeration).

**Fig. 3:**
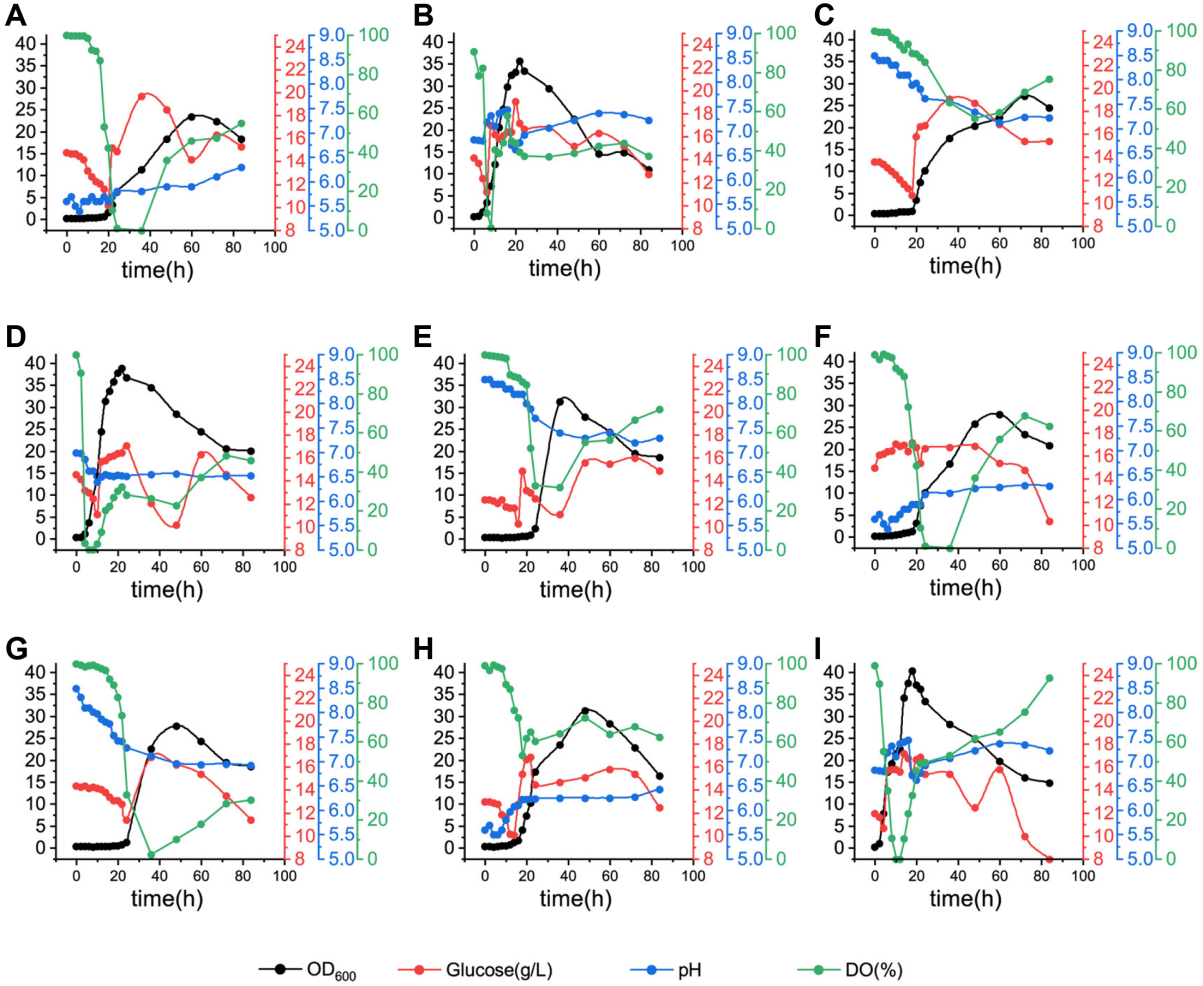
Trends of cell growth, glucose concentration, pH and DO during fed-culture in 300-ml bioreactors. The symbols ‘A-I’ correspond to Run 1-9 in Table 3. (**A**) A1B1C1, (**B**) A1B2C2, (**C**) A1B3C3, (**D**) A2B1C2, (**E**) A2B2C3, (**F**) A2B3C1, (**G**) A3B1C3, (**H**) A3B2C1, and (**I**) A3B3C2. ‘A1, A2, A3’ represented three gradients of culture temperature ranging from 37 to 41°C ‘B1, B2, B3’ represented three gradients of aeration ranging from 0.6 to 2.0 vvm. ‘C1, C2, C3’ represented three gradients of initial pH ranging from 5.5 to 8.5.

The initial pH was the most important factor among these three factors. The mean of MK-7 production at pH 7.0 was 129.55 mg/l, while the mean of the MK-7 output at pH 5.5 was only 59.20 mg/l. It might be related to the fact that the strain BS20DFHG did not grow well when the initial pH was set at 5.5 (Fig. 3). When the initial pH was set at 8.5, the growth of the strain showed a delay of 10 h compared with the strains cultured at pH 7.0 (Fig. 3). The trend of growth curves in all conditions was similar to that in shake flasks. When the fermentation temperature was 41°C, the production of MK-7 was higher than that at the other temperatures, regardless of the other two factors (Table 3). The aeration, which was expected to play an essential role in fermentation [11], however, didn’t have much effect on the biosynthesis of MK-7. As a result, the optimal fermentation scheme could be determined as 41°C, 2 vvm and an initial pH of 7.0 (A3B3C2). Under these conditions, the engineered strain BS20DFHG produced the highest MK-7 titer of 185.57 ± 6.3 mg/l in a 300-ml bioreactor.

### Constructing a Cell Growth Kinetic Model of BS20DFHG in 50-l Bioreactor

**Fig. 4:**
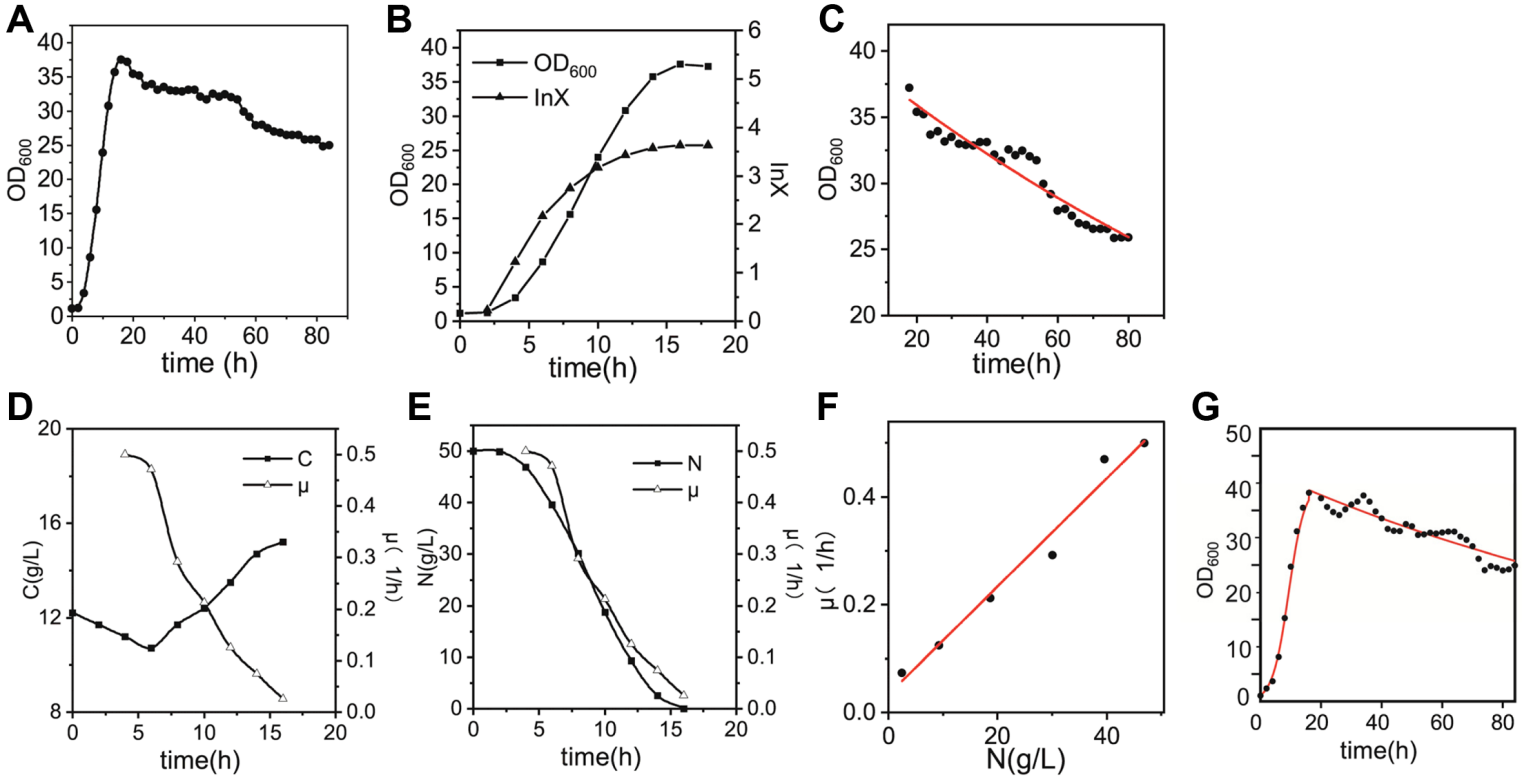
Diagrams of cell growth kinetic model parameters. The ‘μ’ meant the biomass specific growth rate of the strain BS20DFHG, the ‘C’ meant the concentration of carbon source in the bioreactor and the ‘N’ meant the concentration of nitrogen source in the bioreactor. The ‘lnX’ meant logarithm of the biomass ‘X’ with the constant ‘*e*’ as the base. (**A**) Growth curve of the engineered strain BS20DFHG in a 50-l bioreactor. (**B**) The dots presented the growth curves of the engineered strain BS20DFHG in the first 18 h. The triangles meant the values of ‘lnX’. (**C**) The dots showed the value of OD_600_ from 18 h to 84 h. The red curve showed the fitted curve of OD_600_ and time. (**D**) The curve with dots showed the change of the ‘C’ from 0 h to 16 h. The curve with hollow triangle showed the change of the ‘μ’ from 0 h to 16 h. (**E**) The curve with dots showed the change of the ‘N’ from 0 h to 16 h. The curve with hollow triangle showed the change of ‘μ’ from 0 h to 16 h. (**F**) The dots showed the value of the ‘μ’ to different ‘N’ and the red line was the fitted line of ‘μ’ and ‘N’. (**G**) The red line showed the simulation curve of cell growth kinetic model and the black solid dots showed values of the experimental OD_600_ values.

After optimizing the three parameters, we determined the optimal fermentation scheme for MK-7 production. According to the optimization experiments of fermentation parameters such as pH and temperature, the biomass of BS20DFHG at the end of logarithmic growth would affect the final production of MK-7. So, we decided to employ the optimized scheme in 50-l bioreactor fermentation (Fig. S2). Subsequently, a cell growth kinetic model was constructed to describe and predict the cell growth of BS20DFHG. We separated the growth curve into two parts according to the increase or decrease trend of OD_600_. As shown in Fig. 4A, the strain BS20DFHG grew rapidly in the first 18 h, and the OD_600_ slowly decreased in the following 3 days. The growth of the strain BS20DHG could be modeled by an equation in the form of Eq. (1), including the microbial biomass ‘X’, the death coefficient ‘K_d_’ and the specific growth rate ‘μ’. In the first 18 h of the fermentation process (Fig. 4B), the growth curve indicated that there is a relationship between the specific growth rate ‘μ’ and the concentration of limiting nutrients or inhibiting substrates. The negative correlation between ‘μ’ and the concentration of carbon source (C) is presented in Fig. 4D. However, the concentration of glucose (10 g/l) is not considered to inhibit bacterial growth based on previous experimental results (Fig. 2A). Therefore, we turned our attention to the consumption of the nitrogen source which could be seen in Fig. 4E, because the nitrogen source usually affects the synthesis of proteins in cells and further affects the growth of strains. As can be seen in Fig. 4F, the concentration of nitrogen source (N) and the ‘μ’ exhibit positive correlation after linear fitting (Eq. (2)) (A = 0.01, B = 0.0333). The dynamic relationship of the ‘N’ and ‘X’ was given in the form of Eq. (3) (Y_X/N_ = 0.7276).

Due to the rich resources in the medium and the exponential growth of the strain BS20DFHG, we assumed the ‘K_d_’ = 0 during the first 18 h, and the growth of BS20DFHG in the 50-l bioreactor was modeled in the form of Eqs. (1), (2), and (3). In terms of the decline part, Eq. (4) was obtained after integrating Eq. (1) with μ = 0 in the following 3 days. It could demonstrate the decline in the growth of BS20DFHG over time. ‘X_0_’ which was obtained as 40.05 means the initial ‘X’ of the second part and the ‘K_d_’ was recognized as 0.0054 (Fig. 4C). Thus, the dynamic relationship between the ‘μ’, ‘N’ and ‘t’ could be described by Eq. (4). So far, we have constructed a cell growth kinetic model of BS20DFHG in a 50-l bioreactor.

(1) $$\frac{\mathrm{dX}}{\mathrm{dt}}=X*(\mu -K_{d})$$

(2) μ=A*N+B

(3) $$\frac{\mathrm{dN}}{\mathrm{dt}}=\frac{\mathrm{dX}}{\mathrm{dt}}*\frac{1}{Y_{X/N}}$$

(4) $$X=X_{0}*\exp(-K_{d}*t)$$

Next, we carried out fermentation experiments in 50-l bioreactor to verify the accuracy of the model and observed the highest titer of MK-7 as 242 ± 5.5 mg/l. As can be seen from Fig. 4G, the fitted curve was close to the experimental values, indicating that the cell growth kinetic model can describe the actual growth process reasonably well. It was indicated that the concentration of nitrogen was significant for the early growth of the strain BS20DFHG. Increasing the initial concentration of soy peptone, a mixed nitrogen source or fed-addition of nitrogen, can be important strategies to further improve the production of MK-7. The cell growth kinetic model of BS20DFHG cannot explain the MK-7 production directly, but it is a powerful tool to explore the relationship between them. From this kinetic model, more thorough experiments can be designed to explore the relationship between MK-7 production and OD_600_.

In conclusion, after increasing the relative transcription level of *isp*-genes to increase the flux of the MEP pathway, we obtained the highest production of MK-7 as 415 ± 3.2 mg/l in BS20DFHG, a 29% increase compared with BS20. Then we attempted to figure out the crucial factors of the liquid fermentation process and optimized them by parallel bioreactors. Finally, a cell growth kinetic model was developed to demonstrate the growth of BS20DFHG in a 50-l bioreactor accurately. It helps us better understand the growth of BS20DFHG in a bioreactor and guide the next step of optimization, and therefore, the established fermentation process paves the way for the industrial production of MK-7 in the future.

## Supplemental Materials



Supplementary data for this paper are available on-line only at http://jmb.or.kr.

## Figures and Tables

**Table 1:** Strains and plasmids used in this study.

Names	Characteristics	Reference
Strains		
BS20	*Bacillus subtilis* 168 P_veg_ -*kinA*-ΔPAS-A Δ*kinB* ΔspoIIA Δspo0IIE, P_43_-*menF*,P_43_ -*menB* P hbs -*menE*, P_43_ -*entC* ΔdhbB, Phbs -tkt, P_43_-ppsA Δ*ptsG,* P_hbs_ -aroG^fbr^ :: lox72, P_43_ -*aroK* , P_hbs_-*ispA*, P_43_-hepS/T, P _hbs_ -kdpG P_43_-*dxr,* P_43_-*dxs*, P_43_-fni Pmena -*menA* :: lacA, P_mena_-*menA* :: thrC, P_mena_-*menA* :: dacA, P_hag_-Rap60, P_native_-Phr60 :: hag, P_abrb_-*pyk*::pyk, P_abrb_-*uppS*::uppS P_spoiiA_-*ispH*::spoIIE P_spoiiA_-HepS/T::spoIIA	Lab stock
BS20D	BS20 P_43_ -*ispD* :: *ispD*	This study
BS20DF	BS20D P_43_ -*ispF*:: *ispF*	This study
BS20DFE	BS20DF P_43_ -*ispE*:: *ispE*	This study
BS20DFH	BS20DF P_43_ -*ispH*:: *ispH*	This study
BS20DFHG	BS20DFH P_43_ -*ispG*:: *ispG*	This study
Plasmids		
P7C6	Pmd18-T containing *lox71-zeo-lox66* cassette	Lab stock
PDG148	Amp, Km, *E. coli*-*B. subtilis* shuttle vector, containing *cre* under the control of P_spac_	Lab stock

**Table 2:** Experimental factors and levels of fermentation.

Level	A-temperature（°C）	B-aeration（vvm）	C-initial pH
1	33	0.6	5.5
2	37	1.4	7.0
3	41	2.0	8.5

**Table 3:** Effect of three factors on the titer of MK-7.

Run	A	B	C	MK-7 production(mg/l)
1	1	1	1	53.54±2.4
2	1	2	2	90.37±5.3
3	1	3	3	88.73±6.1
4	2	1	2	112.72±5.4
5	2	2	3	136.92±4.9
6	2	3	1	58.75±3.3
7	3	1	3	158.66±6.9
8	3	2	1	65.32±4.1
9	3	3	2	185.57±6.3
Mean1	77.54667	108.3067	59.20333	
Mean2	102.7967	97.53667	129.5533	
Mean3	112.79	111.0167	128.1033	
Range	35.24333	13.48	70.35	
