# Supplementary material for: Combinatorial Methylerythritol Phosphate Pathway Engineering and Process Optimization for Increased Menaquinone-7 Synthesis in Bacillus subtilis
Source: J Microbiol Biotechnol. 2020 Feb 18;30(5):762–9. doi: 10.4014/jmb.1912.12008 (PMC9745656; doi:10.4014/jmb.1912.12008)
Supplement: Supplementary file 1 [file JMB-30-5-762-supple.pdf]

Supplementary Information

Table 1. Primers used in this study

	Sequences (5’-3’)
ispD.F-RT	ATGAGTTATGATGTGGTGATTCCTGCAG
ispD.R-RT	GAAACGGGTAATCGGACAGCAATT
ispF.F-RT	GCATGTCTGGGGAATCGTGAAACA
ispF.R-RT	TTAGCCTTTTTGTATCAGTACTGTCGCCT
ispH.F-RT	ACGTAATTAAAATTTCACCGCGCG
ispH.R-RT	CCGTGCGCAGTGAAAATGACAGTT
ispG.F-RT	GAAATCACACATCGTACAAAAACGCGT
ispG.R-RT	ATGTCAACAACGAGAGGAATGGAAATGC
hbs. F-RT	CAATTATTTTCCGGCAACTGCGTC
hbs. R-RT	GACGCTACAAAAGCAGTTGACTCTG
43D-1. F	GTTTGTACTGTTAAATATACAGGACATACCTTTAATAACAAACG
43D-1. R	GTGAAATTGTTATCCGTGTTTCTTCTCCCTTTACAGCGCCT
43D-2. F	AAAGGGAGAAGAAACACGGATAACAATTTCACACAGGAAACAG
43D-2. R	CATACCACCTATCAAGGGTTTTCCCAGTCACGAC
43D-3. F	ACTGGGAAAACCCTTGATAGGTGGTATGTTTTCGCTTGAACTT
43D-3. R	GCTTTTGCGCTATGATGGTGCA
43F-1. F	GGGAACTCGCCCCTTCTCC
43F-1. R	GTGAAATTGTTATCCGCTCTTACCGCTGAAAGAACTCAATCCACTC
43F-2. F	TGAGTTCTTTCAGCGGTAAGAGCGGATAACAATTTCACACAGGAAA

	C
43F-2. R	TGCACATCAAATCCTTGTCCAATTCTAAACATGTGTACATTCCTCTCT TACCTATAATGGTACC
43F-3. F	ATGTTTAGAATTGGACAAGGATTTGATGTGC
43F-3. R	GATAAAAAAGAACGTACATCACAAGTATGGCCTCAGGCAAAAATGTA TGAA
43F-4. F	TACATTTTTGCCTGAGGCCATACTTGTGATGTACGTTCTTTTTTATCTA TACCAACTGATATACG
43F-4. R	CCGCGACTTATATCGGCATGAG
43E-1. F	AGTTCTGAGAATTGGTATGCCTTATAAGTCCA
43E-1. R	ATTGTTATCCGCTCAGCTTTCACCTACTTCTCCATCTAAACATAAG
43E-2. F	GTAGGTGAAAGCTGAGCGGATAACAATTTCACACAGGAAAC
43E-2. R	TACCACCTATCACGCCAGGGTTTTCCCAGT
43E-3. F	AAACCCTGGCGTGATAGGTGGTATGTTTTCGCTTGAACTTTTAAATAC AGCC
43E-3. R	CGGTATTAACTCGTGCGGATGG
43H-1. F	TCAGCATTATCGGAACAGCCAACT
43H-1. R	GTGAAATTGTTATCCGCTCGAGCGCTTCTCTCTCAGCAGG
43H-2. R	CAGCTCCATTATGTAAGAGCATAAACAGCCTCAGTTTTTTGCTTTTAC TTTTG
43H-3. F	GTAAAAGCAAAAAACTGAGGCTGTTTATGCTCTTACATAATGGAGCT GAACCG

43H-3. R	TTGTACTCTTGACGAATGTGCTGTTC
43G-1. F	GAATATTGCGGGCCAATGTCCC
43G-1. R	AATTGTTATCCGCTCTCGCTCCTTAATTGTAGTTGCCGC
43G-2. F	GAGCGGATAACAATTTCACACAGGAAAC
43G-2. R	TCACTCACTTGCATGTGTACATTCCTCTCTTACCTATAATGGTACC
43G-3. F	AGAGGAATGTACACATGCAAGTGAGTGAAATCACACATCG
43G-3. R	CCTCTGGAACCTTACGGACAATTT

Figure 1. The titers of MK-7 synthesized by strains in shake flasks

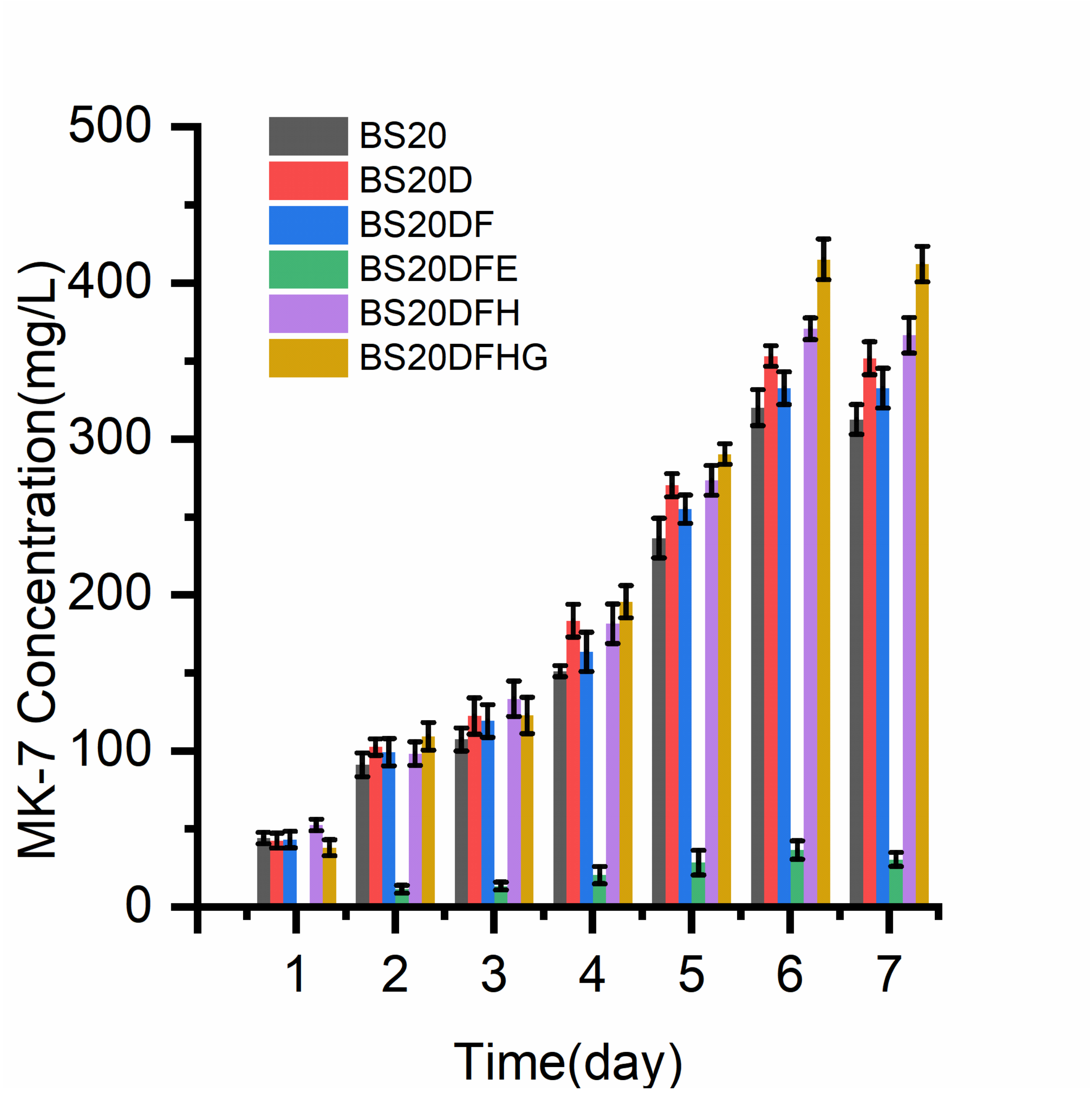

The titers of MK-7 synthesized by strains BS20, BS20D, BS20DF, BS20DFE, BS20DFH and BS20DFHG in 7 days.

Figure 2. The titers of MK-7 synthesized by the strain BS20DFHG in the 50-l fermenter

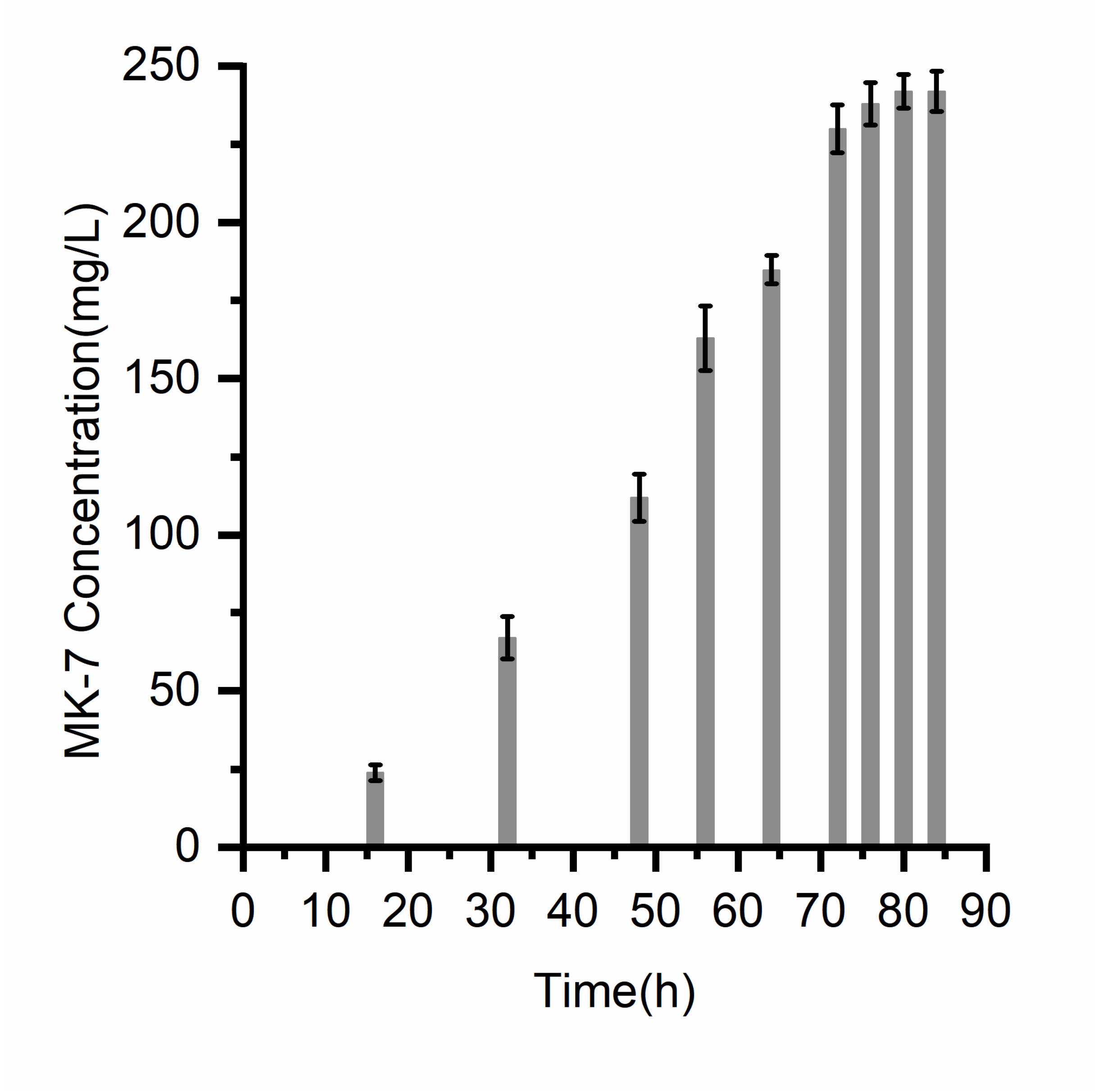

The titer of MK-7 synthesized by the strain BS20DFHG in the 50-l fermenter.

Figure 3. The relative transcriptional levels of *isp*-genes in strains involved

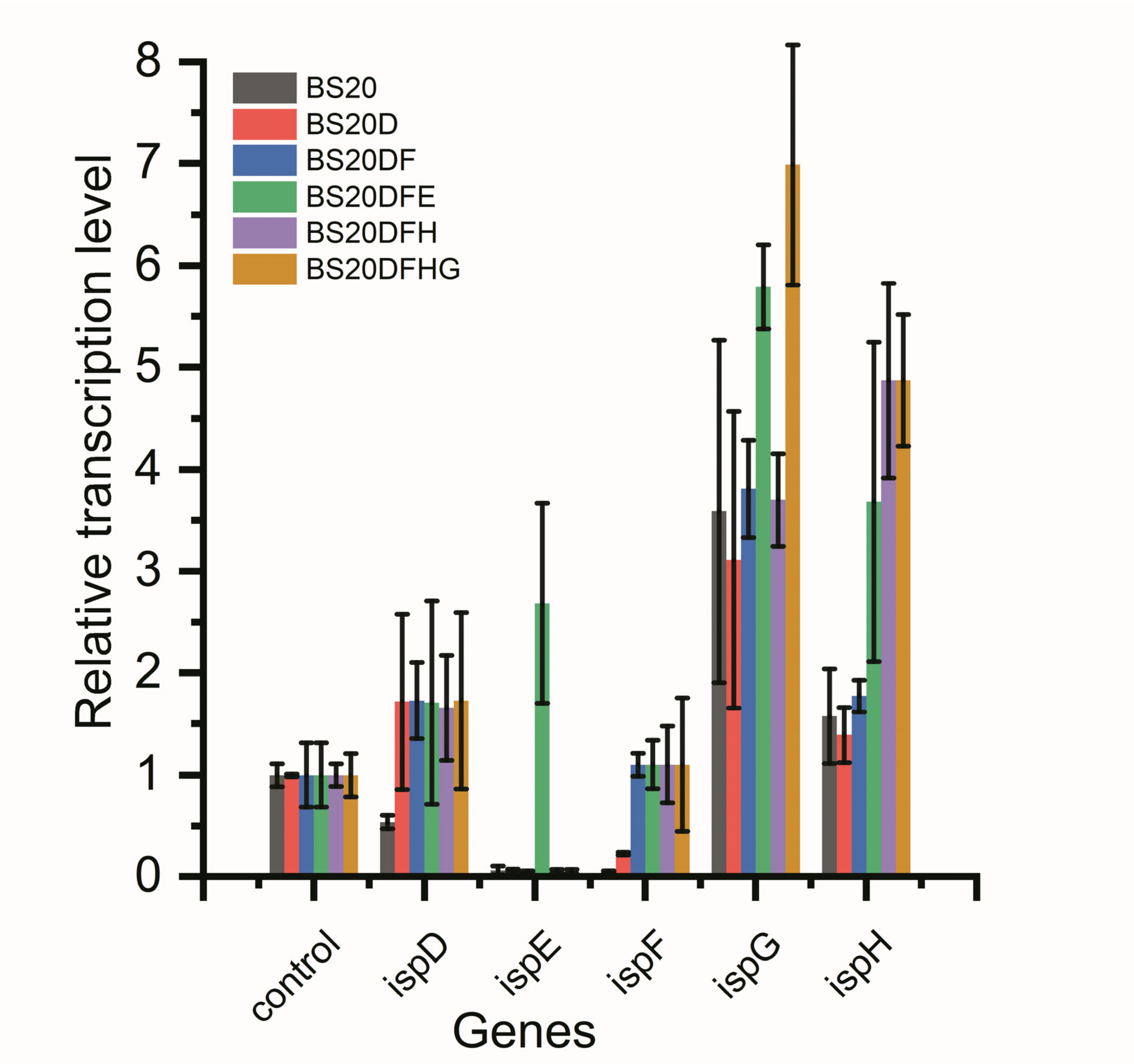

The transcriptional level of the *isp*-genes in BS20, BS20D, BS20DF, BS20DFE, BS20DFH, and BS20DFHG.

Code 1. MATLAB code for cell growth kinetic model simulation

```
function Model_prectc_X
close all

global par

% initia x0 Values
for i = 1:100

    % Km Values
    par.B = 0.0333*rand(1);
    par.Y = 0.7+0.1*rand(1);
    x0(1,1)=1.12; x0(1,2)=50;
    [time,X] = ode23s(@pathw_fun,[0 11.4],x0);

    time_hat = [0:0.1:11.4];
    for k = 1:2
        X_hat(:,k) = interp1(time,X(:,k),time_hat);
    end

X_cella(i,:) = X_hat(:,1)';
X_Na(i,:) = X_hat(:,2)';

```

```
  end % of sampling loop

X_cell1 = mean(X_cella);
X_cell_std = std(X_cella);
X_N = mean(X_Na);
X_N_std = std(X_Na);

time_hat1 = [0:0.14:15.96];
time_hat2 = [16.1:0.14:84];
X2 = 42.642*exp(-0.006*time_hat2);
time_hat = [0:0.14:84];
X_cell = [X_cell1    X2];

figure(1)
subplot(1,2,1)

time_sy=[0,2,4,6,8,10,12,14,16,20,22,24,26,28,30,32,34,36,38,40,42,44,46,48,50,52,54,56,58
,60,62,64,66,68,70,72,74,76,78,80,82,84];
OD_sy=[1.057,2.35,3.67,8.15,15.25,24.7,31.17,35.5,38.25,37.25,35.65,34.7,34.15,35.2,36.09,
36.64,37.75,36.62,34.81,33.55,31.6,31.25,31.2,32.5,32.1,30.5,30.6,30.9,30.76,30.95,31.1,31.1
,30.2,29.6,28.42,24.16,24.05,24.8,24.5,24,24.2,24.9];
```

```
plot(time_hat,X_cell,'r-','LineWidth',2)
set(gca, 'FontSize', 14);
hold on
scatter(time_sy,OD_sy,['k','o'])

axis([0 84 0 50]);
g=['X'];
title(g,'fontsize',12);

subplot(1,2,2)
boundedline(time_hat1,X_N,X_N_std,'r-','alpha');
hold on
plot(time_hat,X_N,'r-','LineWidth',2)
set(gca, 'FontSize', 14);
axis([0 16 0 50]);
g=['N'];
title(g,'fontsize',12);

end

function dx_dt = pathw_fun(~,x)
global par
```

```
% Reaction 2
miu= 0.01*x(2)+par.B;
% Reaction 1
dx_dt(1,1) = x(1)*miu;
% Reaction 3
dx_dt(2,1) = -dx_dt(1,1)/par.Y;

end
```
